# Predictors of noncompliance to pulmonary tuberculosis treatment: An insight from South America

**DOI:** 10.1371/journal.pone.0202593

**Published:** 2018-09-11

**Authors:** Samanta Madeira de Oliveira, Stephan Altmayer, Matheus Zanon, Luzielio Alves Sidney-Filho, Ana Luiza Schneider Moreira, Paulo de Tarso Dalcin, Anderson Garcez, Bruno Hochhegger, José da Silva Moreira, Guilherme Watte

**Affiliations:** 1 Postgraduate program in Respiratory Medicine, Universidade Federal do Rio Grande do Sul, Rio Grande do Sul, Porto Alegre, Brazil; 2 Department of Radiology, Universidade Federal de Ciências da Saúde de Porto Alegre, Rio Grande do Sul, Porto Alegre, Brazil; 3 Department of Surgery, Universidade Federal do Espírito Santo, Vitória, Espirito Santo, Brazil; 4 Department of Respiratory Medicine, Irmandade da Santa Casa de Misericordia de Porto Alegre, Rio Grande do Sul, Porto Alegre, Brazil; 5 Department of Public Health, Universidade do Vale do Rio dos Sinos, Sao Leopoldo, Rio Grande do Sul, Brazil; Indian Institute of Technology Delhi, INDIA

## Abstract

**Purpose:**

To investigate the factors associated with a higher risk of noncompliance to tuberculosis (TB) treatment in Porto Alegre, Brazil.

**Methods:**

We identified 478 adult patients for this case-control study undergoing treatment for confirmed pulmonary TB. Cases (noncompliance) were defined as patients who stopped treatment for more than 30 consecutive days (n = 118). Controls were defined as all patients who completed treatment and were cured (n = 360). Factors associated with noncompliance were calculated with unadjusted and adjusted odds ratio (OR).

**Results:**

The rate of noncompliance in our study was 25%. The factors of noncompliance after adjustments in the overall population were, in order of magnitude, living in an area of lower income (OR = 4.35, 95%CI: 2.50–7.58), abuse of drugs (OR = 2.73, 95%CI: 1.47–5.09), nonadherence to a previous treatment regimen (OR = 2.1, 95%CI: 1.28–3.45), and history of smoking (OR = 1.72, 95%CI: 1.00–3.00). Age, race, gender, level of education, HIV infection or diabetes status were not associated with a higher risk of noncompliance. In the subgroup of re-treatment cases, poverty (OR = 2.65; 95%CI = 1.06–6.66), smoking history (OR = 2.94; 95%CI = 1.09–7.92), male gender (OR = 3.25; 95%CI = 1.32–8.0), and younger age (OR = 4.3; 95%CI = 1.15–16.07) were also associated with a higher risk of dropout.

**Conclusion:**

Predictors of poor compliance to TB treatment were low income, abuse of drugs, re-treatment cases and history of smoking.

## Introduction

Tuberculosis (TB) remains as an important cause of death from an infective agent in the world [1]. Paradoxically, TB is a curable disease in patients who complete the treatment scheme with highly effective antituberculous therapy (ATT) [2,3]. Treatment adherence is essential to cure the disease, minimize the transmission of the bacilli in the community and for the decrease of drug-resistant bacteria [3]. The impact of noncompliance to ATT is serious for both the patients who may need more extended treatment regimens and the public health, since those subjects may continue spreading the disease for longer periods [2–4].

Several strategies have been proposed to improve treatment compliance, such as a fixed-dose combination therapy, directly observed therapy (DOT), and ensuring access to medication at the time of diagnosis [5]. In Brazil, all these strategies are currently part of the government strategy of TB treatment, but the rate of noncompliance remains high [5–7]. Interventions to improve treatment compliance require a better understanding of the individual barriers that can burden the long-term continuation of the medications.[8] Thus, identifying risk factors for treatment discontinuation is essential to determine the subjects that are at a higher risk of noncompliance to create patient-centered interventions to improve adherence and treatment completion.

The goal of this study was to investigate the factors associated with a higher risk of noncompliance to tuberculosis treatment dropout in Porto Alegre, southern Brazil. We further stratified the cases of noncompliance between treatment-naïve patients and re-treatment cases to identify the differences in risk factors between these groups.

## Methods

### Study participants

We retrospectively identified consecutive adult patients referred for pulmonary TB treatment in Porto Alegre, southern Brazil, from January 2014 to December 2014. The city has four referral tuberculosis centers (RTC), and patients from all centers were included in this study. The inclusion criteria were: (1) Age ≥ 18 years; (2) confirmed diagnosis of pulmonary TB (i.e., any person with a positive sputum culture for *Mycobacterium tuberculosis*, or at least two smear-positive results for acid-fast bacilli, or one smear positive for bacilli plus evidence of TB on diagnostic chest x-ray), (3) and treatment with standard antituberculous drug scheme followed in Brazil (rifampin, isoniazid, pyrazinamide, and ethambutol) for at least 6 months. Information regarding recommended routine ATT regimens, duration of treatment, and alternative regimens in consideration of comorbidities is provided in S1 File. Pediatric patients, patients with identified multidrug resistance bacteria (MDR), or those who had undergone alternative treatment regimens due to comorbidities or MDR were not included in the study. The sputum exam was done by concentrated fluorescent microscopy method after culture inoculation [9]. The municipal ethics committee of Porto Alegre and the institutional review board of the Universidade Federal do Rio Grande do Sul approved this study.

We used the official national questionnaire of notifiable disease (S2 File) for identification of patients and data acquisition. Also, all medical records were analyzed in the RTCs of Porto Alegre to gather information on TB symptoms, demographics, and socioeconomic data. Ethical approval was obtained from the governmental review board and the local institutional board review. Each patient was assigned a unique study number for identification. Patient’s names were not used in any study related documents.

Cases (noncompliance) were defined as all confirmed bacteriologically pulmonary TB (criteria above) who had dropped out from the routine treatment scheme. Dropout was considered when the patient did not show up in the last medical office visit and stopped the treatment for at least 30 consecutive days. Controls were defined as all patients with confirmed bacteriologically pulmonary TB (criteria above) who had completed the same treatment and were cured. Cure was defined as two negative smear bacilloscopy (one in the attendance phase and the other at the end of the treatment) or completion of therapy in addition to clinical and radiological recovery in the absence of sputum during the treatment. Both cases and controls were further stratified as “new cases”, those treatment naïve or in-therapy for less than a month; and “re-treatment cases”, individuals who relapsed before complete cure despite of a previous adequate treatment in the last 5 years, or when the patient spontaneously dropped out of treatment after 30 days of therapy and was again referred to restart treatment.

### Definition of variables

The following independent variables were analyzed in this study: sex, age, self-reported ethnicity, level of education (in years), human development index income at the municipal level (HDI-M), homeless status, and presence of other associated health problems such as HIV infection, smoking status, drug use, and diabetes. The income of the participants was defined based according to the location of the basic health unit where the patient was first diagnosed with TB, considering the income dimension of the Human Development Index at the municipal level (HDI-M) of the city of Porto Alegre in the state of Rio Grande do Sul, Brazil. The HDI-M was obtained from the Brazilian Human Development Atlas, produced by the United Nations Development Programme (UNDP).

The HIV seropositivity was confirmed by at least two positive ELISA tests followed by a positive western blot test as confirmation. Tobacco smoking was defined as smoking at least 100 cigarettes in a lifetime. Drug use refers to the harmful or hazardous use of psychoactive substances, not occasionally.

### Statistical analyses

Descriptive statistics were used to describe the characteristics of the total sample and by the cases (tuberculosis treatment dropout) and controls (tuberculosis cure). The distribution of the characteristics was described by absolute and relative numbers between cases and controls, using the Fisher's exact test to explore the heterogeneity of proportions. To investigate the potential factors associated with tuberculosis treatment dropout, an unadjusted and adjusted OR with 95% confidence intervals were calculated by unconditional logistic regression. Wald test for heterogeneity of proportions (categorical variables) and linear trend (ordinal variables) was obtained. Only the characteristics associated with the tuberculosis treatment dropout that showed a p-value lower than 0.20 in unadjusted analysis were considered confounding factors and were included in the multivariable analysis. Additionally, we explored the individual analysis stratified by new cases of tuberculosis and re-treatment cases. A two-tailed statistical significant difference was defined at p<0.05. The data analysis was performed using Stata, version 12.0 (StataCorp LP, College Station, Texas, USA).

## Results

The characteristics of the study subjects are shown in Table 1. A total of 478 subjects, composed of 118 dropout cases and 360 controls, with a mean age of 40.6 ±16.1 years, was included in this study. The incidence of treatment discontinuation was 24.7% (95% CI, 20.8–28.6) in the overall population. Discontinuation of treatment was most common during the first four months of treatment. In the noncompliant group, 23.7% patients discontinued ATT during the first 30 days of treatment, 16.1% during 30–60 days, 12.7% during 60–90 days, and 16.9% during 90–120 days. By the end of the third month, up to 69.4% of noncompliant population had already discontinued treatment. At the end of the 6-month mark, 52.2% of our “controls” were already considered cured and therefore out of the study, while 85.5% of our “cases” were out of the study due to noncompliance.

**Table 1:** Demographic characteristics of the population.

Characteristics	Total Sample (n = 478)	Cases (n = 118)	Controls (n = 360)	*p*-value
Sex				0.085
Female	195 (40.8)	40 (33.9)	155 (43.1)	
Male	283 (59.2)	78 (66.1)	205 (56.9)	
Age (years)				0.016
≤ 30	156 (32.6)	42 (35.6)	114 (31.7)	
31–49	185 (38.7)	54 (45.8)	131 (36.4)	
≥ 50	137 (28.7)	22 (18.6)	115 (31.9)	
Race				<0.001
White	290 (60.7)	52 (44.1)	238 (66.1)	
Non-white	188 (39.3)	66 (55.9)	122 (33.9)	
Education (years)				0.008
≤ 4	122 (25.5)	37 (31.4)	85 (23.6)	
5–10	215 (45.0)	59 (50.0)	156 (43.3)	
≥ 11	141 (29.5)	22 (18.6)	119 (33.1)	
Income^a^				<0.001
0.75 (low)	103 (21.6)	50 (42.4)	53 (14.7)	
0.80	65 (13.6)	13 (11.0)	52 (14.4)	
0.85 (high)	310 (64.9)	55 (46.6)	255 (70.8)	
Homeless				0.028
No	459 (96.0)	109 (92.4)	350 (97.2)	
Yes	19 (4.0)	9 (7.6)	10 (2.8)	
Smoking status				0.002
Non-smoker	324 (67.8)	66 (55.9)	258 (71.7)	
Smoker	154 (32.2)	52 (44.1)	102 (28.3)	
Drug use				<0.001
No	394 (82.4)	78 (66.1)	316 (87.8)	
Yes	84 (17.6)	40 (33.9)	44 (12.2)	
HIV infection				0.014
Positive	359 (75.1)	78 (66.1)	281 (78.1)	
Negative	119 (24.9)	40 (33.9)	79 (21.9)	
Diabetes				0.289
No	446 (93.3)	113 (95.8)	333 (92.5)	
Yes	32 (6.7)	5 (4.2)	27 (7.5)	
Treatment category				<0.001
New	338 (70.7)	63 (53.4)	275 (76.4)	
Re-treatment	140 (29.3)	55 (46.6)	85 (23.6)	

Data is presented as “n” (%)

^a^ HDI-M income: Human Development Index at municipal level.

Discontinuation of treatment was higher among re-treatment cases compared to the treatment-naïve subjects (39.2% vs. 18.6%, p <0.001). Most cases were male (66.1%), were aged 31–49 years (45.8%), non-white (55.9%), had 5–10 years of education (50%), were assisted in an area with higher HDI-M (46.6%), were not homeless (92.4%), were non-smokers (66.1%), HIV positive (66.1%), had no diabetes (95.8%) and were new treatment cases (53.4%). Except for gender (p = 0.08) and diabetes status (p = 0.87), there was a significant difference in all other demographic characteristics between cases and controls. In comparison to controls, the cases were more likely to be from low-income regions (42.4% vs. 14.7%), drug users (33.9% vs. 12.2%), smokers (44.1% vs. 28.3%) and re-treatment cases (46.6% vs. 23.6%). Of note, the proportion of subjects with HIV was lower in the cases when compared to controls (66.1% vs. 78.1%, p = 0.014).

The factors associated with noncompliance to ATT are described in Table 2. Age, race, education, income, smoking and drug use, HIV infection, and treatment category exhibited statistically significant associations with the TB treatment dropout in the unadjusted analysis, but gender and DM status were not. After the adjustments, only income, smoking status, drug use, and treatment category remained associated with treatment dropout. On the other hand, age, race, education, HIV infection lost significance. Discontinuation of treatment was approximately four times higher (OR = 4.35; 95%CI: 2.50–7.58) among the subjects assisted in the areas with the lowest HDI-M income level. Also, noncompliance was 72% higher in subjects who smoke (OR = 1.72; 95%CI: 1.00–3.00), and 173% higher among subjects who used drugs (OR = 2.73; 95%CI: 1.47–5.09). Regarding treatment category, re-treatment cases of tuberculosis exhibited approximately two times (OR = 2.10; 95%CI: 1.28–3.45) higher odds of dropping out from treatment.

**Table 2:** Unadjusted and adjusted odds ratio for the factors associated with noncompliance to antituberculous treatment in Porto Alegre.

Characteristics	Unadjusted OR	*p*-value*	Adjusted OR	*p*-value*
Sex		0.080		0.105
Female	1.00 (ref. cat.)		1.00 (ref. cat.)	
Male	1.47 (0.95, 2.28)		1.50 (0.90, 2.50)	
Age (years)		0.039		0.100
≤ 30	1.93 (1.08, 3.43)		1.78 (0.86, 3.70)	
31–49	2.15 (1.24, 3.75)		1.85 (0.96, 3.59)	
≥ 50	1.00 (ref. cat.)		1.00 (ref. cat.)	
Race (skin color)		<0.001		0.062
White	1.00 (ref. cat.)		1.00 (ref. cat.)	
Non-white	2.48 (1.62, 3.78)		1.54 (0.94, 2.51)	
Education (years)		0.005		0.831
≤ 4	2.35 (1.30, 4.28)		1.00 (0.49, 2.05)	
5–10	2.05 (1.19, 3.53)		1.07 (0.58, 2.00)	
≥ 11	1.00 (ref. cat.)		1.00 (ref. cat.)	
Income^a^		<0.001		<0.001
0.75 (low)	4.37 (2.70, 7.10)		4.35 (2.50, 7.58)	
0.80	1.16 (0.59, 2.27)		1.01 (0.46, 2.21)	
0.85 (high)	1.00 (ref. cat.)		1.00 (ref. cat.)	
Homeless		0.025		0.399
No	1.00 (ref. cat.)		1.00 (ref. cat.)	
Yes	2.89 (1.14, 7.29)		1.83 (0.63, 5.31)	
Smoking status		0.002		
Nonsmoker	1.00 (ref. cat.)		1.00 (ref. cat.)	0.031
Smoker	1.99 (1.30, 3.06)		1.72 (1.00, 3.00)	
Drug use		<0.001		
No	1.00 (ref. cat.)		1.00 (ref. cat.)	0.003
Yes	3.68 (2.25, 6.04)		2.73 (1.47, 5.09)	
HIV infection		0.010		0.475
Positive	1.00 (ref. cat.)		1.00 (ref. cat.)	
Negative	1.82 (1.16, 2.88)		1.08 (0.62, 1.87)	
Diabetes		0.225		
No	1.00 (ref. cat.)		---	
Yes	0.55 (0.21, 1.45)			
Treatment category		<0.001		0.005
New	1.00 (ref. cat.)		1.00 (ref. cat.)	
Re-treatment	2.82 (1.83, 4.37)		2.10 (1.28, 3.45)	

^a^ HDI-M income: Human Development Index at municipal level, considering the location region of the basic health unit in the city.

*p-values of Wald test for heterogeneity of proportions (categorical variables) and for linear trend (ordinal variables).

We stratified the sample into new cases and re-treatment cases to investigate the factors associated with noncompliance to ATT in each category (Table 3). For new cases, being assisted in the regions with the lowest income (OR = 5.57; 95%CI: 2.79–11.13) and drug use (OR = 3.81; 95%CI: 1.71–8.46) were the only factors significantly associated with higher risk of noncompliance in the adjusted analysis. There was a slight trend towards smoking history in the crude analysis, but it was not significant in the adjusted analysis (OR = 1.23; 95%CI: 0.61–2.49). In the re-treatment subgroup, being assisted in the region with lowest income remained associated with dropout (OR = 2.65; 95%CI = 1.06–6.66), so as history of smoking (OR = 2.94; 95%CI = 1.09–7.92), but drug use lost its significance in the adjusted analysis (OR = 1.71; 95%CI = 0.60–4.86). However, two other variables were associated with treatment dropout in this group: being a male (OR = 3.25; 95%CI = 1.32–8.0), younger age (OR = 4.3; 95%CI = 1.15–16.07, for the group ≤30 years), whereas low education level demonstrated a trend towards increased risk of dropout in the adjusted analysis (OR = 3.57; 95%CI = 0.96–13.23).

**Table 3:** Unadjusted and adjusted odds ratio for noncompliance stratified by new cases and re-treatment cases of tuberculosis (n = 118).

Characteristics	New cases of tuberculosis (n = 63)				Re-treatment cases of tuberculosis (n = 55)			
	Unadjusted OR	p-value	Adjusted OR	p-value	Unadjusted OR	p-value	Adjusted OR	p-value
Sex		0.895				0.016		0.009
Female	1.00 (ref. cat.)		---		1.00 (ref. cat.)		1.00 (ref. cat.)	
Male	1.04 (0.60, 1.81)				2.48 (1.18, 5.21)		3.25 (1.32, 8.00)	
Age (years)		0.113		0.523		0.196		0.033
≤ 30	1.87 (0.89, 3.89)		1.23 (0.52, 2.93)		2.00 (0.75, 5.33)		4.30 (1.15, 16.07)	
31–49	1.94 (0.94, 4.00)		1.28 (0.56, 2.92)		2.02 (0.81, 5.01)		4.00 (1.28, 12.49)	
≥ 50	1.00 (ref. cat.)		1.00 (ref. cat.)		1.00 (ref. cat.)		1.00 (ref. cat.)	
Race (skin color)		0.003		0.165		0.036		0.147
White	1.00 (ref. cat.)		1.00 (ref. cat.)		1.00 (ref. cat.)		1.00 (ref. cat.)	
Non-white	2.31 (1.32, 4.02)		1.45 (0.77, 2.71)		2.10 (1.05, 4.20)		1.83 (0.79, 4.22)	
Education (years)		0.423				0.014		0.066
≤ 4	1.30 (0.60, 2.83)		---		4.20 (1.35, 13.05)		3.57 (0.96, 13.23)	
5–10	1.60 (0.84, 3.06)				2.77 (0.93, 8.22)		2.06 (0.60, 7.07)	
≥ 11	1.00 (ref. cat.)				1.00 (ref. cat.)		1.00 (ref. cat.)	
Income^a^		<0.001		<0.001		0.010		0.035
0.75 (low)	4.87 (2.59, 9.16)		5.57 (2.79, 11.13)		2.78 (1.28, 6.04)		2.65 (1.06, 6.66)	
0.80	0.65 (0.22, 1.94)		0.62 (0.18, 2.09)		1.49 (0.56, 3.95)		1.54 (0.50, 4.76)	
0.85 (high)	1.00 (ref. cat.)		1.00 (ref. cat.)		1.00 (ref. cat.)		1.00 (ref. cat.)	
Homeless		0.182		0.546		0.287		
No	1.00 (ref. cat.)		1.00 (ref. cat.)		1.00 (ref. cat.)		---	
Yes	2.70 (0.63, 11.61)		2.37 (0.46, 12.10)		1.96 (0.57, 6.76)			
Smoking status		0.062		0.287		0.010		0.037
Nonsmoker	1.00 (ref. cat.)		1.00 (ref. cat.)		1.00 (ref. cat.)		1.00 (ref. cat.)	
Smoker	1.71 (0.97, 3.01)		1.23 (0.61, 2.49)		2.57 (1.25, 5.27)		2.94 (1.09, 7.92)	
Drug use		<0.001		0.002		0.003		0.333
No	1.00 (ref. cat.)		1.00 (ref. cat.)		1.00 (ref. cat.)		1.00 (ref. cat.)	
Yes	3.53 (1.85, 6.74)		3.81 (1.71, 8.46)		3.48 (1.53, 7.90)		1.71 (0.60, 4.86)	
HIV infection		0.278				0.117		0.413
Positive	1.00 (ref. cat.)		---		1.00 (ref. cat.)		1.00 (ref. cat.)	
Negative	1.43 (0.75, 2.71)				1.76 (0.87, 3.56)		1.33 (0.57, 3.14)	
Diabetes		0.539				0.560		
No	1.00 (ref. cat.)		---		1.00 (ref. cat.)		---	
Yes	0.71 (0.24, 2.12)				0.51 (0.05, 4.99)			

^a^ HDI-M income: Human Development Index at municipal level, considering the location region of the basic health unit in the city.

## Discussion

We have shown that the incidence of noncompliance among treatment-naïve and re-treatment patients referred to TB treatment in Porto Alegre was approximately 19% and 40%, respectively. The factors associated with a higher chance of noncompliance after adjustments were, in order of magnitude, living in an area of lower income, abuse of drugs, cases of re-treatment, and history of smoking. Age, race, gender, level of education, HIV infection or DM status were not associated with a higher risk of noncompliance in the overall population.

In 2008, Porto Alegre had the highest annual incidence of tuberculosis among Brazilian capitals, with a ratio of 101 cases per 100.000 inhabitants [10]. This number is hypothesized to be related to the poverty, high prevalence of HIV infection, and difficulties in the health system [7,10]. Although the adoption of DOT is recommended to all patients subject to TB treatment in Brazil [5], in clinical practice the self-administration remains the standard of care. The patient is usually given a supply of treatment for 30 days and returns to the office on a monthly basis for reassessment and refilling of medications until the end of treatment [7]. Consequently, the observed overall noncompliance incidence of 25% is somehow expected and consistent with the finding of Moro et al [11]. In the same study, patients under DOT had a noncompletion rate of nearly half compared to those not on DOT. Previous studies have demonstrated that treatment dropout occurs in the first three months of treatment [6,7,11]. Thus, although it is difficult to accurately predict which patient is unlikely to adhere, there is a need to identify the risk factors for noncompliance that can aid providers to identify patients at a higher risk of dropping treatment as early as possible [12].

In the present study, there was a clear difference in the risk factors associated with treatment compliance when patients were stratified into “new cases” and “re-treatment cases”. We believe these differences can be used in clinical practice to select which patient would benefit from more closely supervised treatment, such as DOT. In the multivariate analysis of treatment-naïve patients, living in an area of low income and drug use were the only factors significantly associated with a higher risk of dropout in the multivariate. These two variables are consistently reported as risk factors by previous studies [11,13,14]. Other factors such as HIV, age, gender, and ethnicity were not associated with noncompliance, what is also consistent with some reports [11,14], despite some weaker evidence pointing to an association of gender and HIV status to nonadherence [12].

On the other hand, when considering the “re-treatment cases,” three other variables became significantly associated with poor compliance: young age, male gender and low level of education (<4 years). Thus, these factors should be considered by the provider when restarting treatment for a patient with a previous history of relapse, but not for treatment-naïve patients. Drug use was not significantly associated with poor adherence in this group, which could be explained by the lack of power to demonstrate its significance in the subgroup analysis. Similarly, homeless status appeared as a non-significant trend toward a higher risk of dropout in all analysis, possibly due to the low number of homeless people in our cohort. However, previous studies have reported this variable as a risk of noncompliance [11,13,15].

Other variables have been described in the literature as associated with poor compliance. Lack of effective social support networks and unstable living circumstances are unfavorable for adherence [16], whereas family support—including the collection of medication and emotional support to the patient—has a positive impact on adherence [17–19]. Adherence is a complex phenomenon and often requires difficult decisions for a patient to complete a lengthy treatment [15]. Some patients prioritize work over taking treatment, what is more usual in rural areas, since attending a clinic-based treatment, such as DOT, involves spending less time at a labored activity [15,19]. Finally, satisfaction with the health care provider has been shown to be an especially important factor for compliance [20].

The strengths of this work include a large sample of 478 cases and the exploration of specific risk factors associated with poor compliance in the subgroup of patients with a previous history of dropout. Most of the previous works were developed with treatment-naïve patients only or did not specify if re-treatment cases were included [6,7,13,21]. We also have limitations in our study. First, it was not possible to evaluate the reason for noncompliance due to the retrospective nature of the study. Although the assessment of this parameter would be elucidative, it does not hinder the conclusions of the study. Also, we determined the income of the patients based on the area of residency. However, this approach to estimate socioeconomic data has already been validated [22]. Lastly, we did not assess daily compliance to ATT regimen, but we defined noncompliance as failure to refill ATT medications for 30 consecutive days.

## Conclusions

The factors associated with a higher chance of noncompliance to ATT were, after adjustments, living in an area of low income, abuse of drugs, poor compliance to previous ATT regimen, and history of smoking. Among re-treatment patients three other variables were associated with higher risk of noncompliance: young age, being a male, and low education level.

## Supporting information

S1 File Routine antituberculous therapy for pulmonary tuberculosis in Brazil. (PDF)

S2 File Brazilian questionnaire for notification and surveillance of tuberculosis (in Portuguese). (PDF)

S3 File Dataset. (XLSX)
