# Supplementary material for: Predictors of noncompliance to pulmonary tuberculosis treatment: An insight from South America
Source: PLoS One. 2018 Sep 11;13(9):e0202593. doi: 10.1371/journal.pone.0202593 (PMC6133354; doi:10.1371/journal.pone.0202593)
Supplement: S1 File — (PDF) [file pone.0202593.s001.pdf]

S1 File. Routine antituberculous therapy for pulmonary tuberculosis in Brazil

The routine drug regimen for pulmonary tuberculosis in Brazil for most patients ≥10 years of age is an intensive phase of “RHZE” for 2 months (Rifampin 150mg + Isoniazid 75mg + Pyrazinamide 400mg + Ethambutol 275mg – combined in a single pill, once a day)*, followed by continuation phase of “RH” for at least 4 months (Rifampin 150mg + Isoniazid 75mg – combined in a single pill, once a day)*.

*We use the following recommended weight-adjusted dosages: 20-35kg 2 pills/day; 36-50kg 3 pills/day; >50kg 4 pills/day.

Information regarding special regimens for patients with comorbidities (e.g., chronic kidney disease, cirrhosis, and adverse drug-reactions) can be found on the national government guideline (in Portuguese):
http://bvsms.saude.gov.br/bvs/publicacoes/manual_recomendacoes_controle_tuberculose_brasil.pdf (pages 58 – 75) – *last accessed on 7/6/2018*
