# Supplementary material for: Predictors of noncompliance to pulmonary tuberculosis treatment: An insight from South America
Source: PLoS One. 2018 Sep 11;13(9):e0202593. doi: 10.1371/journal.pone.0202593 (PMC6133354; doi:10.1371/journal.pone.0202593)
Supplement: S2 File — (PDF) [file pone.0202593.s002.pdf]

República Federativa do Brasil
Ministério da Saúde

SINAN
SISTEMA DE INFORMAÇÃO DE AGRAVOS DE NOTIFICAÇÃO
FICHA DE NOTIFICAÇÃO / INVESTIGAÇÃO TUBERCULOSE

Nº

CRITÉRIO LABOTORIAL - é todo caso que, independentemente da forma clínica, apresenta pelo menos uma amostra positiva de baciloscopia, ou de cultura, ou de teste rápido molecular para tuberculose.
CRITÉRIO CLÍNICO-EPIDEMIOLÓGICO - é todo caso que não preenche o critério de confirmação laboratorial acima descrito, mas que recebeu o diagnóstico de tuberculose ativa. Essa definição leva em consideração dados clínico-epidemiológicos associados à avaliação de outros exames complementares (como os de imagem, histológicos, entre outros).

Dados Gerais

1 Tipo de Notificação — 2 - Individual

2 Agravo/doença — **TUBERCULOSE** — Código (CID10) A 16. 9

3 Data da Notificação

4 UF

5 Município de Notificação — Código (IBGE)

6 Unidade de Saúde (ou outra fonte notificadora) — Código

7 Data do Diagnóstico

Notificação Individual

8 Nome do Paciente

9 Data de Nascimento

10 (ou) Idade — 1 - Hora 2 - Dia 3 - Mês 4 - Ano

11 Sexo — M - Masculino F - Feminino I - Ignorado

12 Gestante — *1-1ºTrimestre 2-2ºTrimestre 3-3ºTrimestre 4- Idade gestacional Ignorada 5-Não 6- Não se aplica 9-Ignorado*

13 Raça/Cor — *1-Branca 2-Preta 3-Amarela 4-Parda 5-Indígena 9- Ignorado*

14 Escolaridade
0-Analfabeto 1-1ª a 4ª série incompleta do EF (antigo primário ou 1º grau) 2-4ª série completa do EF (antigo primário ou 1º grau)
3-5ª à 8ª série incompleta do EF (antigo ginásio ou 1º grau) 4-Ensino fundamental completo (antigo ginásio ou 1º grau) 5-Ensino médio incompleto (antigo colegial ou 2º grau)
6-Ensino médio completo (antigo colegial ou 2º grau) 7-Educação superior incompleta 8-Educação superior completa 9-Ignorado 10- Não se aplica

15 Número do Cartão SUS

16 Nome da mãe

Dados de Residência

17 UF

18 Município de Residência — Código (IBGE)

19 Distrito

20 Bairro

21 Logradouro (rua, avenida,...) — Código

22 Número

23 Complemento (apto., casa, ...)

24 Geo campo 1

25 Geo campo 2

26 Ponto de Referência

27 CEP

28 (DDD) Telefone

29 Zona — 1 - Urbana 2 - Rural 3 - Periurbana 9 - Ignorado

30 País (se residente fora do Brasil)

Dados Complementares do Caso

Dados complementares

31 Nº do Prontuário

32 Tipo de Entrada — 1 - Caso Novo 2 - Recidiva 3 - Reingresso Após Abandono 4 - Não Sabe 5 - Transferência 6 - Pós-óbito

33 Populações Especiais — 1 - Sim 2 - Não 9 - Ignorado
População Privada de Liberdade
Profissional de Saúde
População em Situação de Rua
Imigrante

34 Beneficiário de programa de transferência de renda do governo — 1 - Sim 2 - Não 9 - Ignorado

35 Forma — 1 - Pulmonar 2 - Extrapulmonar 3 - Pulmonar + Extrapulmonar

36 Se Extrapulmonar — 1 - Pleural 2 - Gang. Perif. 3 - Geniturinária 4 - Óssea 5 - Ocular 6 - Miliar 7 -Meningoencefálico 8 - Cutânea 9 -Laringea 10- Outra_______

37 Doenças e Agravos Associados — 1 - Sim 2 - Não 9 - Ignorado
Aids
Alcoolismo
Diabetes
Doença Mental
Uso de Drogas Ilícitas
Tabagismo
Outras ________________________

38 Baciloscopia de Escarro (diagnóstico) — 1 - Positiva 2 - Negativa 3 - Não Realizada 4 - Não se aplica

39 Radiografia do Tórax — 1 - Suspeito 2 - Normal 3 - Outra Patologia 4 - Não Realizado

40 HIV — 1 - Positivo 3 - Em Andamento 2 - Negativo 4 - Não Realizado

41 Terapia Antirretroviral Durante o Tratamento para a TB — 1 - Sim 2 - Não 9 - Ignorado

42 Histopatologia — 1 - Baar Positivo 2 - Sugestivo de TB 3 - Não Sugestivo de TB 4 - Em Andamento 5 - Não Realizado

43 Cultura
1 - Positivo
2 - Negativo
3 - Em Andamento
4 - Não Realizado

44 Teste Molecular Rápido TB (TMR-TB)
1 - Detectável sensível à Rifampicina
2 - Detectável Resistente à Rifampicina
3 - Não Detectável 4 - Inconclusivo
5 - Não Realizado

45 Teste de Sensibilidade
1 - Resistente somente à Isoniazida
2 - Resistente somente à Rifampicina
3 - Resistente à Isoniazida e Rifampicina
4 - Resistente a outras drogas de 1ª linha
5 - Sensível 6 - Em andamento 7 - Não realizado

46 Data de Início do Tratamento Atual

47 Total de Contatos Identificados

Município/Unidade de Saúde — Cód. da Unid. de Saúde

Nome — Função — Assinatura
